# Autophagy and intermittent fasting: the connection for cancer therapy?

**DOI:** 10.6061/clinics/2018/e814s

**Published:** 2018-11-27

**Authors:** Fernanda Antunes, Adolfo Garcia Erustes, Angélica Jardim Costa, Ana Carolina Nascimento, Claudia Bincoletto, Rodrigo Portes Ureshino, Gustavo José Silva Pereira, Soraya Soubhi Smaili

**Affiliations:** I Departamento de Farmacologia, Escola Paulista de Medicina, Universidade Federal de Sao Paulo (EPM-UNIFESP), Sao Paulo, SP, BR; II Departamento de Ciencias Biologicas, Universidade Federal de Sao Paulo, Diadema, SP, BR

**Keywords:** Apoptosis, Autophagy, Fasting, Cancer, Therapy

## Abstract

Cancer is a leading cause of death worldwide, and its incidence is continually increasing. Although anticancer therapy has improved significantly, it still has limited efficacy for tumor eradication and is highly toxic to healthy cells. Thus, novel therapeutic strategies to improve chemotherapy, radiotherapy and targeted therapy are an important goal in cancer research. Macroautophagy (herein referred to as autophagy) is a conserved lysosomal degradation pathway for the intracellular recycling of macromolecules and clearance of damaged organelles and misfolded proteins to ensure cellular homeostasis. Dysfunctional autophagy contributes to many diseases, including cancer. Autophagy can suppress or promote tumors depending on the developmental stage and tumor type, and modulating autophagy for cancer treatment is an interesting therapeutic approach currently under intense investigation. Nutritional restriction is a promising protocol to modulate autophagy and enhance the efficacy of anticancer therapies while protecting normal cells. Here, the description and role of autophagy in tumorigenesis will be summarized. Moreover, the possibility of using fasting as an adjuvant therapy for cancer treatment, as well as the molecular mechanisms underlying this approach, will be presented.

## Autophagy: definition and mechanisms

The 2016 Nobel Prize in Physiology or Medicine was awarded to Yoshinori Ohsumi for his initial elucidation of the morphological and molecular mechanisms of autophagy in the 1990s 1,2. Autophagy is an evolutionarily conserved lysosomal catabolic process by which cells degrade and recycle intracellular endogenous (damaged organelles, misfolded or mutant proteins and macromolecules) and exogenous (viruses and bacteria) components to maintain cellular homeostasis 3,4. The specificity of the cargo and the delivery route to lysosomes distinguishes the three major types of autophagy. Mircroautophagy involves the direct engulfment of cargo in endosomal/lysosomal membrane invaginations 5. Chaperone-mediated autophagy (CMA) recycles soluble proteins with an exposed amino acid motif (KFERQ) that is recognized by the heat shock protein hsc70; these proteins are internalized by binding to lysosomal receptors (LAMP-2A) 6. Macroautophagy (herein referred to as autophagy) is the best-characterized process; in this process, cytoplasmic constituents are engulfed within double-membrane vesicles called autophagosomes, which subsequently fuse with lysosomes to form autolysosomes, where the cargo are degraded or recycled 3,7. The degradation products include sugars, nucleosides/nucleotides, amino acids and fatty acids that can be redirected to new metabolic routes for cellular maintenance 8-10.

Autophagy occurs at basal levels under physiological conditions and can also be upregulated in response to stressful stimuli such as hypoxia, nutritional deprivation, DNA damage, and cytotoxic agents 11,12. The molecular machinery that mediates the autophagic process is evolutionarily conserved in higher eukaryotes and regulated by specific genes (ATG genes), which were initially characterized in yeast 13,14. Each stage is controlled by different protein complexes regulated by the activation or inactivation of several stress-responsive pathways, such as those involving mammalian target of rapamycin (mTOR—nutrient), AMP-activated protein kinase (AMPK—energy) and hypoxia-inducible factors (HIFs—stress) 3,15. Regarding initialization, the activation of the ULK1 complex (ULK1/2, Atg13, FIP200 and Atg101) signals for autophagosome nucleation under the control of the PI3K III complex (PI3KIII, Beclin-1, Atg14/Barkor, Vps15 and Ambra-1), whose activation induces PIP3 (phosphatidyl inositol 3 phosphate) production, which in turn recruits other Atg proteins to form the phagophore 16. Subsequently, two ubiquitin-like conjugation systems mediate the recruitment of ATG12–ATG5 and microtubule-associated protein light chain 3 (LC3) proteins to the phagophore, allowing its expansion and closure to form the mature autophagosome 17. This process leads to the conversion of the soluble protein LC3-I via conjugation to phosphatidylethanolamine to form an LC3-II membrane-associated form in the cytosol, specifically in the inner and outer membranes of the autophagosome 18,19. Furthermore, LC3-II can interact with adaptor proteins such as p62 (also known as sequestosome-1/SQSTM1), which directs cargo delivery to autophagosomes for further degradation in lysosomes, the final step of autophagy 20,21.

Throughout the past decade, autophagy has attracted considerable attention as a potential target of pharmacological agents or dietary interventions that inhibit or activate this process for several human disorders, including infections and inflammatory diseases 22, neurodegeneration 23, metabolic and cardiovascular diseases 24, obesity 25 and cancer 26,27.

## Autophagy and cancer

The role of autophagy in cancer is complex, and its function may vary according to several biological factors, including tumor type, progression stage and genetic landscape, along with oncogene activation and tumor suppressor inactivation 26,28. Thus, autophagy can be related either to the prevention of tumorigenesis or to the enabling of cancer cell adaptation, proliferation, survival and metastasis 29,30. The initial indication that autophagy could have an important role in tumor suppression came from several studies exploring the essential autophagy gene BECN1, which encodes the Beclin-1 protein, in different cellular models. Liang et al. 31 demonstrated that BECN1 was frequently monoallelically deleted in ovarian, breast and testicular cancer. Moreover, mice harboring allelic loss of BECN1 had a partial autophagy deficiency and were prone to the development of hepatocarcinoma and lung tumors at an advanced age 32,33. However, BECN1 is located adjacent to the well-known tumor suppressor gene BRCA1, which is commonly deleted in hereditary breast cancer. These deletions are generally extensive and affect BRCA1 along with several other genes, including BECN1, suggesting that the deletion of BRCA1, not the deletion of BECN1, is the driver mutation in breast cancer 34. However, autophagy impairment due to a mosaic deletion of ATG5 induces benign liver tumors, demonstrating that different tissues have different responses to autophagy impairment 35. Furthermore, the activation of oncogenes (e.g., PI3KCA) and inactivation of tumor suppressors (e.g., PTEN and LKB1) are associated with autophagy inhibition and tumorigenesis 36. In general, studies from animal models note that the tumor suppressor function of autophagy is associated with cell protection from oxidative stress, DNA damage, inflammation and the accumulation of dysfunctional organelles. Collectively, these phenomena are important factors that could trigger genomic instabilities leading to tumor development 29,37,38. However, the loss of function of autophagy genes has not yet been identified and demonstrated in humans, raising doubts about the relevance of autophagy to tumor initiation in different types of cancer 26. In addition, the autophagic machinery is not a common target of somatic mutations, indicating that autophagy may have a fundamental role in the survival and progression of tumor cells 39.

Once the tumor is established, the main function of autophagy is to provide a means to cope with cellular stressors, including hypoxia, nutritional and growth factor deprivation and damaging stimuli, thus allowing tumor adaptation, proliferation, survival and dissemination 40. Autophagy, by degrading macromolecules and defective organelles, supplies metabolites and upregulates mitochondrial function, supporting tumor cell viability even in constantly stressful environments 11,29. Studies have demonstrated that autophagy increases in hypoxic regions of solid tumors, favoring cell survival. The inhibition of autophagy leads to an intense induction of cell death in these regions 41,42. Moreover, tumors frequently have mutations or deletions in the tumor suppressor protein *p*53, which also favors autophagy induction to recycle intracellular components for tumor growth 43. Although the basal autophagy rate is generally low in normal cells under physiological conditions, some tumors show a high level of basal autophagy, reinforcing the prosurvival role of autophagy in cancer 40,44. RAS-transformed cancer cells undergo autophagy upregulation to supply metabolic needs and maintain functional mitochondria, which in turn favors tumor establishment 45-47. Autophagy also has a supportive role in metastasis by interfering with epithelial-mesenchymal transition constituents to favor tumor cell dissemination 30. Finally, studies have demonstrated that autophagy is commonly induced as a survival mechanism against antitumor treatments, such as chemotherapy, radiotherapy and targeted therapy, contributing to treatment resistance 48,49.

## Autophagy and cancer therapeutics

Because autophagy can inhibit tumor development or favor tumor growth, progression, invasion and treatment resistance, researchers proposed that autophagy modulation could be a new therapeutic strategy in the treatment of some malignancies 28,49,50.

Recently, we published a review on autophagy and cancer, suggesting that some challenges, such as the incomplete understanding of the relationship between autophagy, tumor resistance, and cell death, as well as the identification of new druggable targets, need to be overcome with the aim of pharmacologically modulating autophagy for cancer treatment 51. Some of these suggestions are based on the current literature and on previous studies published by our group demonstrating that combining different agents such as selumetinib and cytarabine with autophagy inhibitors (bafilomycin A1, chloroquine or 3-methyladenine) enhanced the activity of selumetinib and cytarabine against colorectal cancer cells 52 and leukemia cells 53, respectively. Autophagy was also observed in melanoma cells under treatment with palladium complex drugs 54, indicating the importance of investigating the relationship between autophagy and apoptosis during new drug development. Additionally, other studies demonstrated that inhibiting autophagy by chloroquine in combination with sorafenib in an *in vitro* model of glioblastoma 55 and in combination with temozolomide in melanoma patients augmented antitumor treatment efficacy 56. The inhibition of autophagy was also demonstrated to potentiate the response to radiotherapy in ovarian 57 and esophageal cancer 58. The efficacy of autophagy in favoring cell death has been demonstrated in many other cancer models, such as breast cancer, leukemia, prostate cancer, and myeloma 48,49. However, to date, clinical trials have not demonstrated that autophagy inhibition associated with anticancer therapy provided reliable therapeutic benefits to patients 59. Currently, protocols targeting autophagy induction instead of autophagy blockade are under intense investigation in oncology 28,50,60. Nevertheless, no drug currently licensed by any regulatory agency was developed for autophagy modulation, although several approved agents indeed modulate autophagy to some extent 61,62.

## How does dietary restriction modulate autophagy and cancer therapy?

In preclinical studies, dietary restriction (DR) has been shown to extend the lifespan and reduce the development of age-related diseases such as diabetes, cancer, and neurodegenerative and cardiovascular diseases 63. DR promotes metabolic and cellular changes in organisms from prokaryotes to humans that allow adaptation to periods of limited nutrient availability 64. The main changes include decreased blood glucose levels and growth factor signaling and the activation of stress resistance pathways affecting cell growth, energy metabolism, and protection against oxidative stress, inflammation and cell death 64,65. Nutrient starvation also activates autophagy in most cultured cells and organs, such as the liver and muscle, as an adaptive mechanism to stressful conditions 11,66.

Studies demonstrate that dietary interventions can reduce tumor incidence and potentiate the effectiveness of chemo- and radiotherapy in different tumor models, highlighting dietary manipulation as a possible adjunct to standard cancer therapies 63,65. Among the many diet regimens that have been assessed, caloric restriction (CR) and fasting are the methods under intense investigation in oncology 63,65,67. CR is defined as a chronic reduction in the daily caloric intake by 20-40% without the incurrence of malnutrition and with the maintenance of meal frequency 68. In contrast, fasting is characterized by the complete deprivation of food but not water, with intervening periods of normal food intake. Based on the duration, fasting can be classified as (i) intermittent fasting (IF—e.g., alternate day fasting (≥16 hours) or 48 hours of fasting/week) or (ii) periodic fasting (PF—e.g., a minimum of 3 days of fasting every 2 or more weeks) 65. In this article, we do not review CR studies that have been reviewed elsewhere 63,68,69; instead, we focus on studies using IF protocols as an adjuvant to cancer treatment in animals and humans.

Recently, studies in *in vitro* and *in vivo* models have shown that intermittent fasting improved the chemotherapeutic response to cisplatin, doxorubicin, cyclophosphamide 70, oxaliplatin 71, sorafenib 72, mitoxantrone 73, gemcitabine 74, etoposide 75, temozolomide 76 and tyrosine kinase inhibitors 77 in models of glioma, neuroblastoma, melanoma, fibrosarcoma and breast cancer, colon cancer, pancreatic cancer, hepatocellular cancer and lung cancer. IF has also been shown to improve the radiosensitivity of glioma 76 and breast cancer 78 in mice. Interestingly, fasting in combination with cytotoxic agents elicited differential responses in normal and cancer cells, a phenomenon known as differential stress resistance (DSR). For DSR, normal cells prioritize maintenance pathways and inactivate growth factor signaling when nutrients are absent. In contrast, cancer cells, due to oncogene activation, do not inhibit stress resistance pathways, thus becoming vulnerable to cytotoxic treatment 70,75. IF, by reducing the circulating glucose levels, protected mice from doxorubicin toxicity and particularly promoted cardioprotection mediated in part by EGFR1-dependent transcriptional regulation of atrial natriuretic peptide and B-type natriuretic peptide in heart tissue 79. As demonstrated by Tinkum et al. 80, IF also facilitated DNA repair activation mechanisms and preserved small intestinal (SI) stem cell viability as well SI architecture and barrier function after exposure to high-dose etoposide, suggesting that fasting can be applied to reduce side effects and toxicity in patients undergoing chemotherapy.

Although the results of combining IF with anticancer drugs are encouraging, the molecular mechanisms are not completely clear. Lee et al. 81 demonstrated that IF (48-hour fasting) reduced the glucose and IGF-1 levels by 60% and 70%, respectively, in a breast cancer animal model. In a colon cancer model, IF inhibited tumor growth without causing permanent weight loss and decreased M2 polarization of tumor-associated macrophages in mice. *In vitro* data showed autophagy induction and CD73 downregulation, followed by a decrease in extracellular adenosine and the inhibition of M2 polarization due to the inactivation of JAK1/STAT3 82.

When IF cycles were combined with chemotherapy, tumor growth was slowed and overall survival was prolonged in breast cancer, melanoma and neuroblastoma animal models 70. The *in vitro* data showed that this therapeutic combination resulted in increased Akt and S6 kinase phosphorylation, caspase-3 cleavage and apoptosis induction in cancer cells but not in normal cells 70. Other studies demonstrated that the combination of IF and oxaliplatin also reduced tumor growth and glucose uptake *in vivo* and resulted in downregulated aerobic glycolysis followed by augmented oxidative phosphorylation, leading to increased oxidative stress, decreased ATP synthesis and cell death in colon cancer cell models 71. Furthermore, Our group also demonstrated that nutritional deprivation enhanced the sensitivity of both wild type and BRAF^V600E^ human melanoma cells to cisplatin treatment followed by ROS production and mitochondrial perturbation leading to apoptosis without autophagy involvement in the cell death process 83. Pietrocola et al. 73 showed that IF improved the chemotherapeutic response to mitoxantrone and oxaliplatin in murine fibrosarcoma, reducing tumor growth in immunocompetent mice. This group also showed that the impairment of tumor growth was dependent on the cellular immune system as well as on autophagy; IF + chemotherapy could not impair tumor growth in either athymic nu/nu mice or tumor cells after autophagy deficiency was induced by Atg5 knockdown.

The combination of IF and tyrosine kinase inhibitors such as erlotinib, gefitinib, lapatinib, crizotinib and regorafenib promoted the sustained inhibition of the MAPK pathway, leading to antiproliferative effects in breast, colorectal and lung cancer cell models, as well as to the inhibition of tumor growth in an *in vivo* model of lung cancer 77. The combination of IF and the multi-tyrosine kinase inhibitor sorafenib exhibited an additive effect in inhibiting hepatocarcinoma cell proliferation and glucose uptake as well as downregulating the MAPK pathway and the gene expression of BIRC5, DKK1, TRIB3 and VEGF, which are commonly altered in hepatocarcinoma cells 72. In pancreatic cancer, fasting increased the uptake of gemcitabine due to enhanced levels of its transporter (hENT1), thus potentiating cell death. In a xenograft pancreatic cancer model, fasting cycles and gemcitabine treatment induced a reduction in tumor growth of more than 40% 74.

A small pilot study comprising 10 patients diagnosed with breast, prostate, esophageal or lung cancer in advanced stages suggested that periods of intermittent fasting before and after chemotherapy reduces the self-reported side effects of therapy, especially those associated with the gastrointestinal system, as well as weakness and fatigue. Additionally, no negative effect on the chemotherapy response or persistent weight loss was observed 84,85. In another clinical trial, the combination of IF and platinum-based chemotherapy promoted pathologic complete or partial radiographic responses in the majority of patients affected by different stages and types of tumors, such as ovarian, uterine, breast and urothelial cancer. A reduction in leukocyte DNA damage, in addition to decreased levels of circulating IGF-1, has also been reported 86. Both studies established the feasibility of IF in humans and suggested that combining IF with cytotoxic agents in the clinical context is safe and may be well-tolerated by patients, although this regimen may be psychologically uncomfortable for some individuals 84-87. Currently, other clinical trials involving IF combined with chemotherapy in cancer patients are underway; these trials are summarized in Table 1. The results of these trials will be essential for a better evaluation of the clinical potential and application of this new therapeutic strategy.

**Figure 1:**
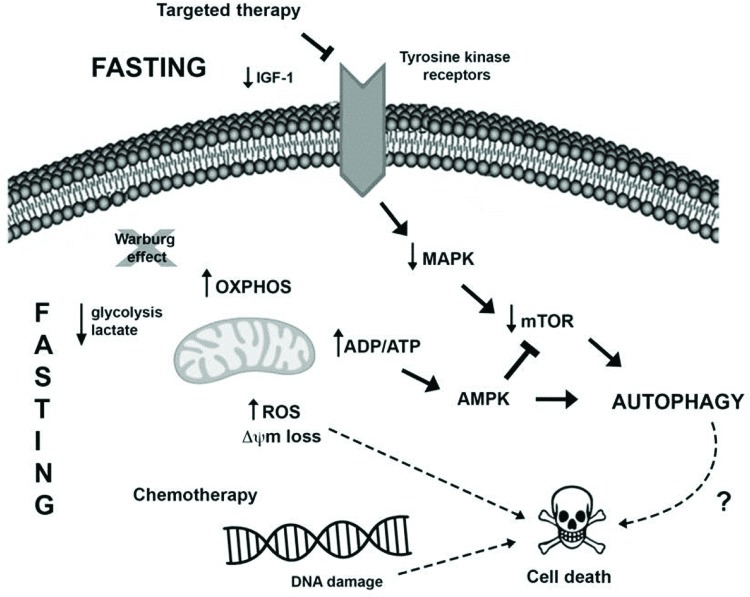
Presumable molecular mechanisms induced by fasting and anticancer treatment to promote intracellular changes and autophagy induction in tumor cells. I) Fasting may oppose the Warburg effect (glucose breakdown by glycolysis even in the presence of oxygen), favoring oxidative phosphorylation in tumor cells and resulting in increased ROS production and reduced levels of lactate and possibly ATP. The increase in the ADP/ATP ratio can activate the AMPK pathway, leading to autophagy induction. Moreover, the sustained stressful environment can result in cell death induction. II) Several tumors harbor mutations that favor MAPK pathway hyperactivation, which enables tumor cell growth, survival and proliferation. Therapies targeting this pathway, as well as fasting, may result in the downregulation of this pathway alongside a reduction in AKT and mTOR activation, resulting in autophagy induction and cell death. III) Furthermore, fasting potentiates the detrimental effects of chemotherapy, such as DNA damage, thus activating the cell death machinery, deregulating pro- and antiapoptotic proteins, and inducing mitochondrial alterations and caspase activation, which in turn culminates in apoptosis.

Another novel pharmacological therapeutic strategy currently being investigated to treat cancer is the combination of caloric restriction mimetics (CRMs) with cytotoxic agents. CRMs are compounds that have different chemical structures and mimic the biochemical and functional effects of CR, such as the activation of AMPK and inhibition of mTOR leading to autophagy induction, the depletion of acetyl-CoA and ATP, and the reduced utilization of glucose, without eliciting the discomfort of CR 88. Several studies demonstrated the tumor-suppressive effects of CRM agents, for example, 2-deoxy-glucose 89, metformin 90,91, mTOR inhibitors 92, resveratrol 73,93, hydroxycitrate 73, spermidine 73,94 and natural compounds such as curcumin 95, in combination with antitumor treatments in different cancer models. The possible connections between fasting and anticancer therapy potentiation in tumor cells are summarized in Figure 1.

In this review, we highlighted the concepts of autophagy, especially in relation to tumorigenesis, as well as the potential of autophagy as a therapeutic target in the treatment of different malignancies. We also pointed out the possibility of using dietary manipulation as an autophagy modulator as well as a cost-effective intervention to increase therapeutic response in the challenging oncologic arena. Furthermore, fasting may protect normal cells from the toxicity of anticancer agents, reducing side effects in patients and increasing the detrimental effects of chemotherapy, radiotherapy and targeted therapy on tumor cells. However, additional studies are required to better understand the molecular mechanisms evoked by fasting, aiming to identify the context in which fasting may be beneficial as an adjunct to cancer treatment. Moreover, further knowledge may also lead to the development of novel pharmacological protocols that replicate effects similar to those of fasting and are more suitable for different oncologic patients.

## AUTHOR CONTRIBUTIONS

Antunes F contributed to the design of the study, wrote most of the study and edited the manuscript. Erustes AG, Costa AJ, Nascimento AC and Trindade CB wrote the manuscript. Ureshino RP, Pereira GJ and Smaili SS wrote, designed and coordinated the study and edited and reviewed the final version of the manuscript. All authors reviewed and approved the final version of the manuscript.

## Figures and Tables

**Table 1:** Completed and current clinical trials investigating the effects of fasting as adjunct therapy to anti-cancer treatment.

Cancer/Phase	Treatment	Outcome/Status	Reference
Breast Cancer, Hormone-resistant Prostate Cancer, Recurrent Prostate Cancer	Chemotherapy + low-calorie diet	Currently recruiting participants	NCT01802346
Advanced Metastatic Prostate Cancer	Chemotherapy + fasting and nutritional therapy	Currently recruiting participants	NCT02710721
HER2 Negative Breast Cancer	Chemotherapy + fasting mimicking diet	Currently recruiting participants	NCT02126449
Breast Cancer	Chemotherapy + short-term fasting (IF)	IF associated with chemotherapy was well tolerated, reduced hematological toxicity in HER2-negative BC patients and also induced a faster recovery of DNA damage in PBMCs (peripheral blood mononuclear cells)	NCT01304251 (96)
Gynecological cancer disease (ovarian and breast cancer)	Chemotherapy + short-term fasting	Completed, no results reported	NCT01954836
Breast cancer	Chemotherapy + short-term fasting	Completed, no results reported	NCT02379585
Malignant Neoplasm	Short-term fasting prior to systemic chemotherapy	Active	NCT01175837
Malignant Neoplasm	Chemotherapy + fasting	Completed, no results reported	NCT00757094
